# Use of Informational Brochures on Knowledge of Cataracts in Rural Ecuador

**DOI:** 10.7759/cureus.35555

**Published:** 2023-02-27

**Authors:** Damla Oncel, Sila Bal, Nataly Karina Cargua Mazon, Zachary Smith, Sarah Marjane, Eileen Gable

**Affiliations:** 1 Ophthalmology, Loyola University Chicago Stritch School of Medicine, Chicago, USA; 2 Ophthalmology, Massachusetts Eye and Ear Infirmary, Boston, USA; 3 Health, Partners for Andean Community Health, Riobamba, ECU; 4 Ophthalmology, Loyola University Medical Center, Chicago, USA

**Keywords:** brochure, health literacy, ecuador, health education, cataract

## Abstract

Background

Cataracts are the leading global cause of preventable blindness. Despite the high prevalence of cataracts in rural Ecuadorian communities, no community-wide educational efforts on the impact of cataract-related blindness have been attempted. This study used an educational brochure to assess individual knowledge of cataract blindness before and after the distribution of the brochure.

Methods

We conducted electronic surveys with 100 patients over the age of 18 who visited the Fundacion Internacional Buen Samaritano Paul Martel (FIBUSPAM) clinic serving the Chimborazo region of Ecuador. Participation in the study included an introduction and written consent followed by a pre-survey. Every patient was given a brochure. After reviewing the brochure, patients were then asked to complete the same survey again. Each survey question received one mark. Knowledge was considered "good" if the subject correctly answered four out of seven questions and "poor" if they scored ≤3.

Results

Of the 100 patients, 21 had poor knowledge surrounding cataracts. Cataract awareness was lowest in the group without formal education (50%). In addition, 17 participants demonstrated poor knowledge before the informational brochure was distributed, and all improved to "good" knowledge after. Following brochure distribution, there was an improved knowledge of cataract anatomy (correct answer: 32.9% to 94.6%), treatment of cataracts (80% to 95.9%), cataract symptoms (36.7% to 95.9%), age at risk (88.8% to 97.3%), and relationship to blindness (93.5% to 98.6%). In contrast, overall knowledge of the risk factors for cataracts (46.8% to 37%) and prevention of the onset of the cataract (81.3% to 77%) slightly decreased after the brochure was given. Overall, the increase in the number of correct answers after the brochure was not significant (p = 0.25).

Conclusions

To our knowledge, this is a rare study to assess the effect of informational brochures on cataract knowledge in rural Ecuador. This study was limited by selection bias and did not look at the long-term recall of knowledge. The results of this study suggest that brochures can improve health awareness; however, brochures alone may not be enough. Additional assessments on the use of oral and visual aids are needed. Health education efforts should go beyond simple brochures and focus on innovative strategies to improve health education and communication efforts.

## Introduction

Vision impairment is a major public health concern globally. Estimates by researchers like Bourne showed that over 1.7 million people will suffer from vision loss by 2025 [1,2]. Cataracts remain the leading cause of blindness in many developing countries and account for over 50% of global cases of blindness [3]. The etiology of cataract formation is multifactorial including maternal nutrition, infection, age, traumatic injuries, ultraviolet radiation, chemical injuries, systemic diseases, endocrine disease, drugs, alcohol use, smoking, and primary ocular diseases [4]. The pathophysiology of this eye disease is due to degenerative processes that denature the lens proteins, ultimately leading to cataract formation [5].

There is a lack of published information on the causes of blindness in Ecuador and Latin America; however, Cass et al. showed that among the causes of unilateral blindness, cataract was the most common cause [6]. One study showed that patients presenting to eye clinics in Ecuadorian communities had high rates of cataracts, and the same study concluded that cost and access were the main barriers to care for this disease [7].

In addition to cost and access issues, a main barrier in many regions of the world is the overall poor health literacy of these communities. Indeed, low health literacy in adults has been associated with increased hospitalizations and increased emergency department visits [8]. To our knowledge, no studies have assessed the effect of community-based health education interventions in Ecuador on patients’ knowledge of eye diseases. The primary objective of this study is to determine and compare the patient’s knowledge of the cataract before and after being given an educational brochure.

## Materials and methods

This was an experimental cross-sectional study of knowledge of cataracts in rural Ecuadorian communities. The inclusion criteria for the participants were that they had to be above 18 years old and visiting the FIBUSPAM (Fundacion Internacional Buen Samaritano Paul Martel) clinic in the Riobamba region of Ecuador over the period between July 22, 2022, and August 13, 2022. Those patients who were not capable of giving informed consent and those younger than 18 years old were excluded from the study. A one-time electronic survey was utilized using a convenience sample of patients recruited through FIBUSPAM medical personnel. The survey is given in the Appendix. The electronic survey had two parts. The first part included demographic details such as age, gender, and education level as well as the history of cataract or family members diagnosed with cataract. The second part had eight questions that evaluated the awareness and knowledge of cataract. Knowledge was graded as good or poor according to summative patient scores. Each question received one point; knowledge was considered good if the subject scored four out of seven (the first question was excluded) and considered poor if the score was 3 or less.

Having heard of the eye disease in question was defined as awareness, and having some understanding of the disease was defined as knowledge. The electronic surveys were translated into both Kichua and Spanish. After the patients completed the surveys, they received an electronic educational brochure and were asked to review it. After review, the patients once again answered the same survey. Survey results were analyzed, and percentages were calculated for cataract awareness in patients grouped according to their educational level. Primary statistical analysis was performed using an independent T-test with Statistical Package for the Social Sciences (SPSS) software, version 18.0 (SPSS Inc., Chicago, IL) to determine whether the educational intervention increased knowledge on cataract, and a p-value was calculated.

## Results

**Figure 1:**
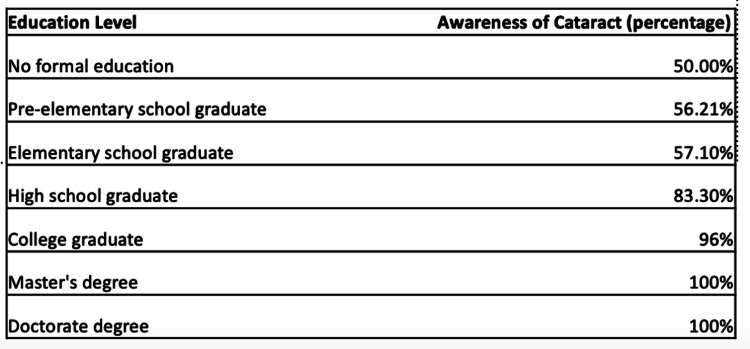
Awareness of cataract in patients according to their educational level groups

Surveys were completed by 100 patients presenting to the FIBUSPAM clinic, out of which 59% were men, and 41% were women. The mean age was 40.73 (range: 19-77 years). According to the education level data analysis, out of 100 participants, 1% graduated with a doctorate degree, 1% graduated with a master’s degree, 27% graduated from college, 42% graduated from high school, 7% graduated from elementary school, 16% graduated pre-elementary school, and 6% did not go to school. Ninety-two percent of the patients were not diagnosed or treated for cataracts before. Fifty-six percent of the patients did not have any family members who were diagnosed with cataract. Out of 100 participants, 21 patients were not aware that they had cataracts. The awareness of cataract was lowest in the group that had no schooling at 50%. The awareness of cataract in the groups that graduated from pre-elementary school and elementary school was 56.25% and 57.1%, respectively. The awareness of cataract in the group that graduated from high school and college was 83.3% and 96.3%, respectively (Figure 1).

**Figure 2:**
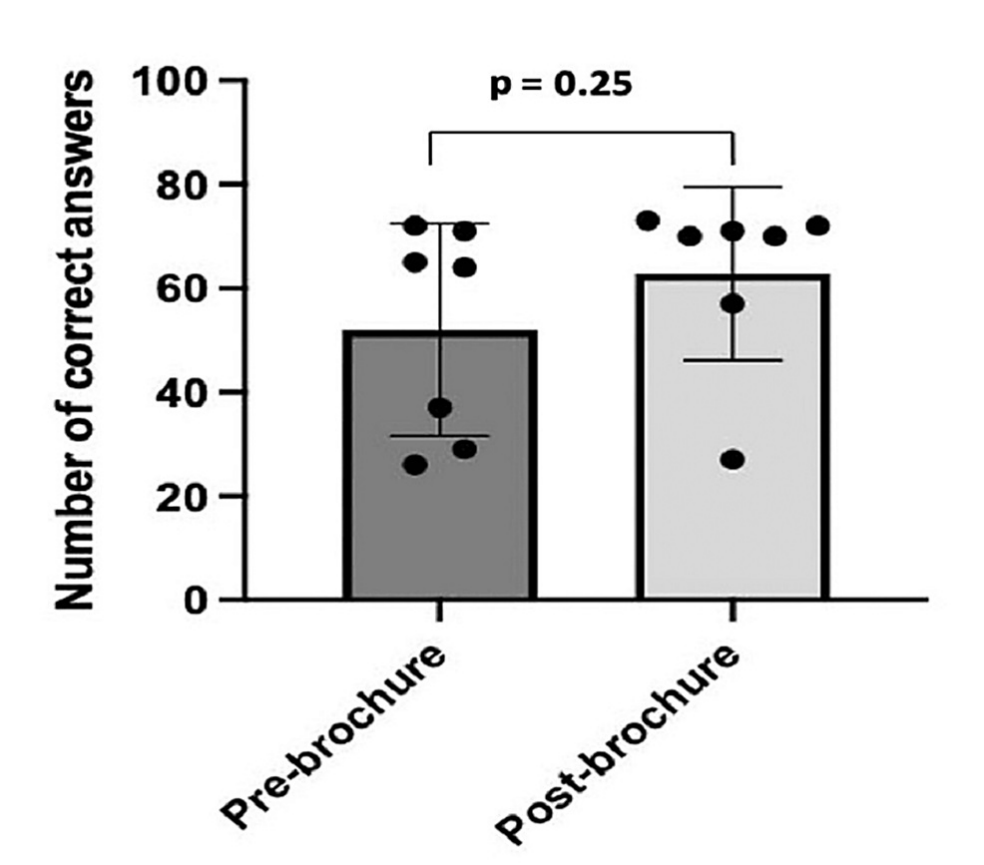
Number of correct answers given to the survey questions that were completed before and after the brochure

Before the informational brochure, only 32.9% of the patients understood that cataracts were an eye disease that affects the lens. Of these patients, 27.8% thought cataracts affected the retina, and 39.2% thought cataracts affected the cornea. After the informational brochure was given, the percentage of patients that knew cataracts affected the lens increased to 94.6%. Eighty percent of the patients knew that cataracts are treated with surgery, and 36.7% knew that symptoms of cataracts include blurry/reduced vision. Both numbers increased to 95.9% following the distribution of the brochure. Approximately 88.8% of the patients knew that age could be a risk factor for cataract formation; after the brochure, this number increased to 97.3%. Furthermore, 93.5% of the patients knew cataracts can lead to blindness; after the brochure, this number increased to 98.6%. Eighty-one percent of the patients knew that the onset of cataracts can be delayed by preventing risk factors, and this number decreased to 77%. Lastly, 46.8% knew that sunlight, diabetes, and smoking are risk factors for cataracts, and this number decreased to 37%. In total, 17 participants had poor knowledge (score of three or less) before the informational brochure was given. After the brochure was distributed, every participant had an overall "good" score (scored at least four questions). Overall, the increase in the number of correct answers after the brochure was not significant (p = 0.25) as shown in Figure 2.

## Discussion

Previous studies showed that access and cost are the major barriers to cataract-related care in Ecuador [7]. Health literacy has a huge impact on health access, with a strong correlation between low health literacy and poor knowledge of access to preventative care services and information [9]. Improved health literacy can drastically reduce health disparities. This study aimed to observe the effect of education on the knowledge of patients. To our knowledge, research on health literacy and its impact on health outcomes is limited in Ecuador [10]. We hoped to illustrate the importance of health education and encourage health literacy efforts in rural Ecuador.

We found that 21% of the patients had no knowledge of cataracts, and this number was highest in those groups that did not have any education, demonstrating that patients with lower education backgrounds had lower health literacy in comparison to those patients who had a higher education background; this comparison has been shown in other studies as well [11]. After patients received the informational brochure on cataracts, there was a significant improvement in knowledge of anatomy, treatment, and symptoms of cataracts as well as risk factors and the relationship between blindness and cataracts. In contrast to these questions, the overall patient knowledge on the risk factors of cataracts and prevention of the onset of the cataract slightly decreased after the brochure was given. This could be due to the design of the brochure, which did not have images associated with every text. Patients who are visual learners could have picked up the information that had images associated with them more effectively. When compared to pre-brochure survey results, every patient in the post-brochure survey had at least four correct answers, corresponding to good knowledge.

To our knowledge, this was the first study to assess the effect of informational brochures on cataract knowledge in rural Ecuador. This study had limitations that should be mentioned. First, the surveys were done on patients that came to the FIBUSPAM clinic; thus, this study was limited by selection bias. Also, the improvement of knowledge was tested immediately after reading the brochure, so this study does not show long-term retention of knowledge.

## Conclusions

In summary, our results suggest that creating educational brochures is helpful to improve health knowledge, whereas informational brochures did not improve knowledge in the last two questions. This may be due to the fact that some patients rely on oral communication rather than written due to poor literacy rates. Therefore, health education efforts should go beyond creating brochures and focus on creating innovative ideas and strategies to improve health education and communication efforts. Further studies should establish best practices that will help patients understand their medical conditions in rural areas in Ecuador.

## Appendices

Survey for cataract knowledge

First Part

Gender: M/F

Age: ___

Education level: ___

Have you been diagnosed and/or treated for cataract before?

(a) Yes

(b) No

Did any family members were diagnosed with cataract?

(a) Yes

(b) No

Second Part

1. Have you heard of cataract?

(a) Yes

(b) No

NOTE: If answered “No,” you can stop the survey here.

2. Cataract is an eye disease that affects which part of the eye?

(a) Retina

(b) Lens

(c) Cornea

3. What is the treatment for cataract?

(a) Glasses

(b) Surgery

(c) Eyedrops

(d) It is not treatable

4. What are the symptoms of cataract?

(a) Fever

(b) Pain in the eye

(c) Blurry/reduced vision

(d) Itchy eye

5. Can increasing age be the risk factor for cataract?

(a) Yes

(b) No

6. Can cataract cause blindness?

(a) Yes

(b) No

7. Can the onset of cataract be delayed by preventing risk factors?

(a) Yes

(b) No

8. What are the risk factors of cataract?

(a) Sun exposure

(b) Diabetes

(c) Smoking

(d) All of the above
